# Pneumatic Bionic Hand with Rigid-Flexible Coupling Structure

**DOI:** 10.3390/ma15041358

**Published:** 2022-02-13

**Authors:** Chang Chen, Jiteng Sun, Long Wang, Guojin Chen, Ming Xu, Jing Ni, Rizauddin Ramli, Shaohui Su, Changyong Chu

**Affiliations:** 1 School of Mechanical Engineering, Hangzhou Dianzi University, Hangzhou 310018, China; hdsgwy@163.com (L.W.); chenguojin@163.com (G.C.); xumzju@163.com (M.X.); nijing2000@163.com (J.N.); messhhui@163.com (S.S.); kevin@hdu.edu.cn (C.C.); 2 Department of Mechanical and Manufacturing Engineering, Faculty of Engineering and Built Environment, University Kebangsaan Malaysia, Bangi 43600, Malaysia; rizauddin@ukm.edu.my

**Keywords:** soft gripper, liquid silicone rubber, rigid-flexible coupling, mechanical modeling, motor micropump

## Abstract

This paper presents a rigid-flexible composite of bionic hand structure design scheme solution for solving the problem of low load on the soft gripping hand. The bionic hand was designed based on the Fast Pneumatic Network (FPN) approach, which can produce a soft finger bending drive mechanism. A soft finger bending driver was developed and assembled into a human-like soft gripping hand which includes a thumb for omnidirectional movement and four modular soft fingers. An experimental comparison of silicone rubber materials with different properties was conducted to determine suitable materials. The combination of 3D printing technology and mold pouring technology was adopted to complete the prototype preparation of the bionic hand. Based on the second-order Yeoh model, a soft bionic finger mathematical model was established, and ABAQUS simulation analysis software was used for correction to verify the feasibility of the soft finger bending. We adopted a pneumatic control scheme based on a motor micro-pump and developed a human–computer interface through LabView. A comparative experiment was carried out on the bending performance of the finger, and the experimental data were analyzed to verify the accuracy of the mathematical model and simulation. In this study, the control system was designed, and the human-like finger gesture and grasping experiments were carried out.

## 1. Introduction

The development of traditional rigid robots is in the relatively early stages; it can complete precise operations, liberate heavy labor and inject new vitality into human production activities [1,2]. For rigid grips regarding items with complex surface shapes and fragile items, the gripping force is difficult to accurately control, and it is easy to cause damage to the surface of the item [3]. Due to its own flexibility, environmental adaptability and interactive safety features, the soft gripping hand can realize functions that are difficult to meet with traditional rigid manipulators. This mechanism is widely used in medical rehabilitation [4], aerospace and disaster relief [5,6,7,8].

At present, the soft gripper driver has received extensive attention from many researchers [9]. Liu Yonggan et al. [10] developed a lightweight, soft manipulator with continuous controllable stiffness based on McKibben pneumatic artificial muscles, which can continuously adjust the stiffness at the required position and allow additional stiffness. Zhu et al. [11] embedded an elastic tube between two layers of soft fabric by sewing, developing fluidic fabric muscle sheets (FFMS) driven by fluid pressure. Marchese et al. [12] proposed an elastic fluid bending drive unit with a bidirectional symmetrical cylindrical elastic air chamber and developed a two-dimensional flexible mechanical arm based on the drive unit. Katia Bertoldi’s team, inspired by octopus tentacles, proposed a cone-shaped elastic air chamber soft driver with a vacuum suction cup that can grab objects of different shapes and materials. Guoliang Zhong et al. [13] proposed a novel soft pneumatic dexterous gripper with fingers made of silicone rubber with pleated channels to make the grasping process more flexible. Shufeng Tang et al. [14] designed a robotic hand whose fingertips are made of hollow rubber to enhance gripping stability. Liu, Shoufeng et al. [15] proposed a dual-module pneumatic actuator with a variable chamber height. The actuator has compliance and passive adaptability, which can increase the contact area and ensure good grasping reliability. Low, Jin Huat et al. [16] proposed a 3D printed soft gripper with embedded sensors to improve the gripping operation and provide sensor feedback. Deimel, R et al. [17] designed a new type of compliant under-actuated soft bionic hand whose shape can be adapted to the shape of specific objects and can achieve complex grasping behavior through a relatively simple control. Y Zhao et al. [18] designed a large-variable rigidity flexible continuum driver based on interference, which can realize large load grasping. Ge L et al. [19], based on the design of soft fabric pneumatic actuators (SFPAs), designed three structures of thumb abduction, finger bending and finger extension and integrated SFPAs into a portable control system. Yangqiao Lin et al. [20] designed a faster response soft hand, which has both fast response and precise control capabilities. Roth J et al. [21] treated the surface of silicone rubber with oxygen and nitrogen plasma and then functionalized it with polyethylene maleic anhydride to improve permanent adhesion. Lamblet M et al. [22] achieved enhanced silicone rubber–acrylic interface bond strength by treating the PDMS matrix. Elsayed Y et al. studied three kinds of silicone rubber materials and derived a constitutive model, and verified the performance of the three materials based on the simulation analysis of the soft air cavity [23,24].

Due to their own advantages, software soft grippers have attracted the attention of researchers and have broad prospects for development. These grippers are constantly improving in the direction of tactile, force feedback and rapid response precision control. Compared with the traditional rigid gripping hand, the soft gripping hand is more adaptable, but its softness will cause the problem of requiring a low load in the gripping hand [25].

Therefore, in this paper, we propose a rigid-flexible coupling pneumatic soft human-like finger to overcome these problems. Based on the principle of a “fast pneumatic network” (FPN) and simple design, a rigid-flexible coupling structure of the pneumatic soft human-like flexible gripping hand is designed. The performance of the finger is tested on silicone rubber materials in order to determine suitable types. The motion mode of the soft bionic hand is similar to that of a human hand, including the rotation and bending functions of the thumb and the other four-finger multi-joint motion functions. By combining 3D printing technology, we used mold pouring technology to complete the prototype preparation of the bionic hand. On the basis of the second-order Yeoh model, the soft bionic finger statics mathematical model was established and ABAQUS simulation analysis software was used for correction to verify the feasibility of the soft human-like finger control system. The control system adopts the scheme of the air pump solenoid valve pneumatic control system and the motor micro pump control system to cooperate with each other and develop human–computer interaction software in the LabView program. Comparative experiments on the bending performance of soft human-like fingers were also carried out to verify the accuracy of the mathematical model and the bionic hand grasping and gesture movements. In this study, we explore the feasibility of rigid-flexible composite human fingers and their further research prospects.

The design method proposed in this paper has the characteristics of simple preparation, flexible grasping and safety. We combine the advantages of rigid materials and flexible materials to carry out research on structural design and performance simulation, prototype preparation, control system development and experimental verification. This research can promote the practical application of the software bionic hand in the fields of life services and medical services and can provide a reference for future research.

## 2. The Structure Design of the Soft Gripper

### 2.1. Structure Design of Soft Finger Driver

**Figure 1:**
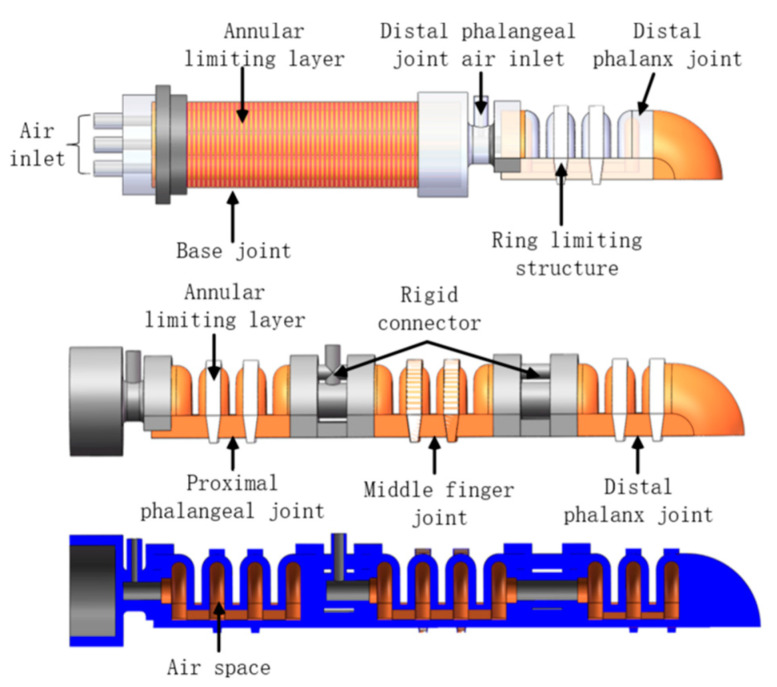
Schematic diagram of soft finger structure.

The soft finger is divided into two parts, the thumb and the other four fingers, which are mainly composed of the base joint and the ring-shaped limiting layer. The limiting layer is used to restrict the radial deformation of the soft driver, the phalangeal joint and the ring-shaped restriction structure and the rigid connector. Figure 1 show that the rigid connector and the ring-shaped limiting layer are made of 3D printed material, and the soft air cavity is made of silicon rubber. Silicone rubber has a mature mechanical simulation analysis model, and it is a hyperelastic material suitable for making soft fingers.

**Figure 2:**
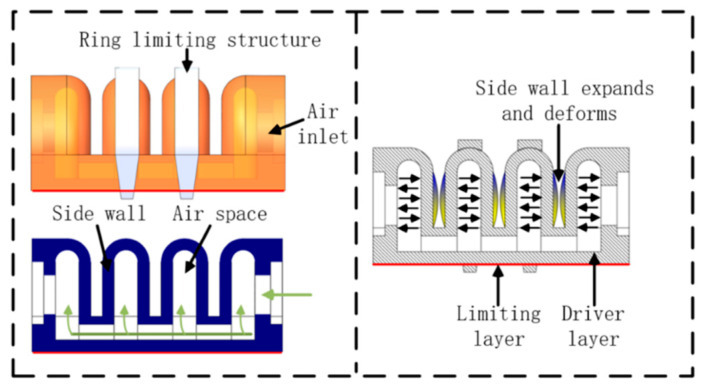
Schematic diagram of the drive structure.

In this paper, the bionic finger soft-driven joint is designed by adopting the Fast Pneumatic Grid Structure (FPN) design, as shown in Figure 2. The FPN structure expands through the sidewalls, which will cause the actuator to bend and deform. The soft-driven joint is mainly composed of multiple air chambers. When the external positive air pressure acts on the inner wall of the air chamber, it will cause the expansion and deformation of the air chamber wall, which will increase the length of the drive layer. Since the bottom confinement layer is not scalable, the entire drive is bent.

Under the action of external air pressure, the top wall and side wall of the driving joint will expand and deform at the same time. However, the expansion and deformation of the top wall is interference deformation. In order to solve this problem, a composite rigid ring confinement structure was adopted, in which the design can be connected and fixed for the top wall of the driving joint air chamber with the bottom of the driver. The ring-shaped restricting structure material is acrylonitrile butadiene styrene (ABS) plastic, which will not be deformed significantly under the action of force, and restrict the outward expansion of the top wall of the air chamber. The flexible ultra-thin steel plate at the bottom can increase the lateral load force of the drive.

**Figure 3:**
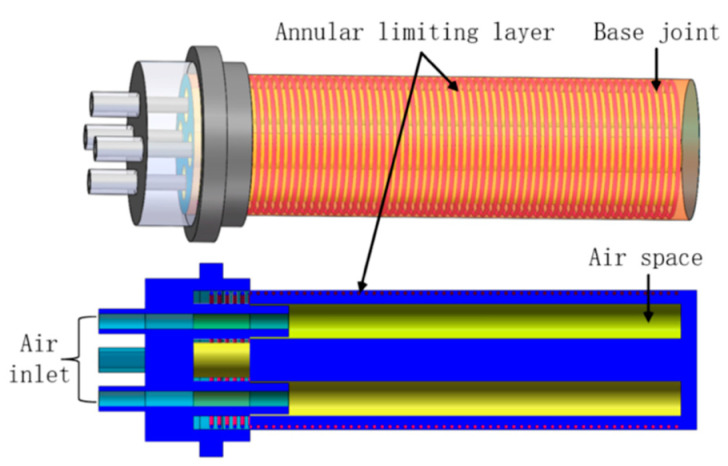
Schematic diagram of base joint structure.

The base joint was designed to achieve the circumferential swing of the thumb. The structure and principle of the base joint are shown in Figure 3. The outer contour of the base joint is cylindrical, and the four-chamber channel structure is designed in a 90° diagonal distribution along the axial model. The outside of the main body adopts fiber spiral winding to limit radial deformation. Under the action of external pressure load, the driving air cavity and the bottom produce a local strain difference effect to realize bending motion.

### 2.2. Production of Soft Gripper

The soft bionic gripper is made of silicon rubber material E60 whose specific performance is shown in Table 1. Silicone rubber was chosen because of its high tensile strength, high elasticity and aging resistance, in addition to its low viscosity and high fluidity. The mixing ratio is 1:1, which is convenient for precisely controlling the silica gel mixing ratio.

**Figure 4:**
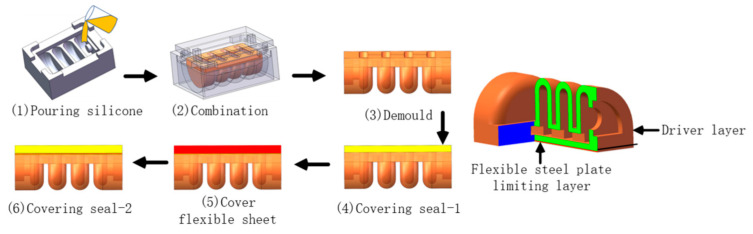
Soft driver production process.

We analyzed the drive joint structure and then formulated a molding production plan, as shown in Figure 4.

The sequence of the production of the soft bionic gripper are as follows: (1) Prepare the drive layer, (2) Cover the sealing layer 1, (3) Place the flexible steel plate, (4) Cover the sealing layer 2; the preparation of the drive joint is completed.

**Figure 5:**
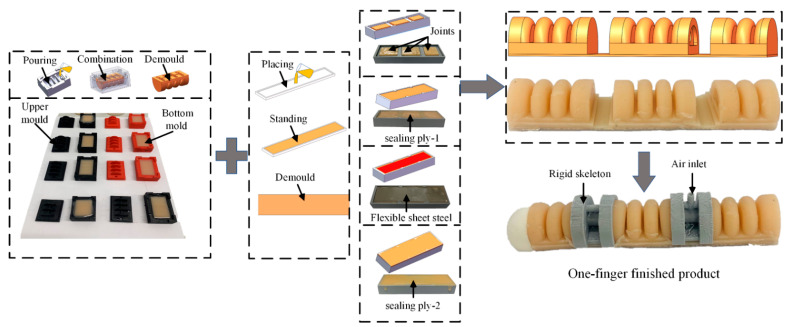
Single finger production process.

**Figure 6:**
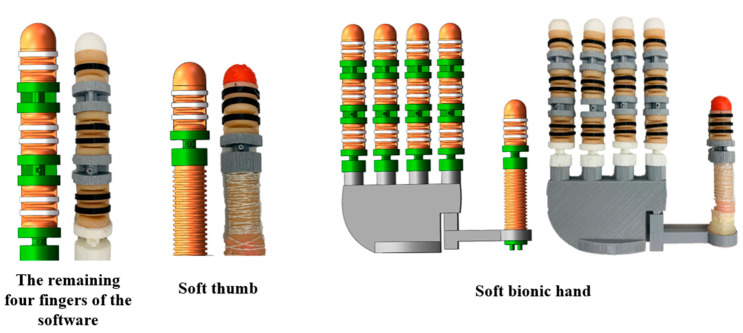
Soft gripper combination.

The mold and rigid skeleton are 3D printed, as shown in Figure 5. The production process is briefly described as follows: silica gel mixing—standing defoaming—pouring into the mold—curing and demoulding—embedding support—gluing assembly. The palm contains the remaining four fingers and thumb connection structure, and the complete assembled soft gripper is shown in Figure 6. The internal hexagonal control method between the thumb joint and the palm structure allows the thumb to have three rotational positions to enhance the flexibility of the thumb movement.

The rigid connection component connects the remaining four fingers of the soft with the palm of the hand through a spin-on component. The palm has a hollow structure, which can reserve space for subsequent finger access to the sensor feedback system.

## 3. Rigid-Flexible Coupling Bionic Hand Mechanics Modeling and Simulation Analysis

### 3.1. Statics Analysis of Soft Human-like Fingers

The second-order Yeoh model is used to establish a soft bionic finger statics mathematical model, which is based on the following assumptions:

(1) Silicone rubber materials are homogeneous and incompressible;
(2) The restrictive layer used is not extensible;
(3) The width and height of the drive remain unchanged under the action of the confinement layer.

The Yeoh model of the second-order strain energy density function is:

(1) $$w=C_{10}\left(I_{1}-3\right)+C_{20}{\left(I_{1}-3\right)}^{2}$$

where w is material strain energy,$\;I_{1}$ is deformation tensor first invariant, $C_{10}$ is the first parameter of the second-order Yeoh model, $C_{20}$ is the second parameter of the second-order Yeoh model.

According to the isotropic hypothesis of rubber theory [26], we can determine the relationship between the strain energy density function ω and the three strain invariants $I_{1},\;I_{2},\;I_{3}$ of the deformation tensor.

(2) $$w=w\left(I_{1},I_{2},I_{3}\right)=w\left(\lambda_{1},\lambda_{2},\lambda_{3}\right)$$

The volume change ratio of the rubber material before and after deformation is 1, that is:

(3) $$I_{3}=\lambda_{1}^{2}\lambda_{2}^{2}\lambda_{3}^{2}=1$$

In the uniaxial tensile test, assuming that the one direction is the main tensile direction, $\lambda_{2}=\lambda_{3}$, $\lambda_{1}=\lambda$. Substitution formula (3):

(4) $$\lambda_{1}=\lambda =\lambda_{2}^{-2}=\lambda_{3}^{-2}$$

According to the theory of the relationship between Cauchy–Green strain and Piola–Kirchhoff stress tensor, the principal stress of silicone rubber material can be obtained:

(5) $$\sigma_{Ti}=2\left(\lambda_{i}{}^{2}\frac{\partial w}{\partial I_{1}}-\frac{1}{\lambda_{i}^{2}}\frac{\partial w}{\partial I_{2}}\right)+P_{e}\left(i=1,\;2,\;3\right)$$

where $\sigma_{Ti}$ is principal stress in all directions, $P_{e}$ is hydrostatic pressure, w is strain energy density function, $I_{i}$ is the strain invariant.

By subtracting the principal stresses in three directions, the hydrostatic pressure can be eliminated $P_{e}$. We are able to obtain the principal stress differences in three directions of the rubber-elastomer:

(6) $$\{\begin{array}{c} \sigma_{T1}-\sigma_{T2}=2\left(\lambda_{1}^{2}-\lambda_{2}^{2}\right)\left(\frac{\partial w}{\partial I_{1}}+\lambda_{3}^{2}\frac{\partial w}{\partial I_{2}}\right)\\ \sigma_{T2}-\sigma_{T3}=2\left(\lambda_{2}^{2}-\lambda_{3}^{2}\right)\left(\frac{\partial w}{\partial I_{1}}+\lambda_{1}^{2}\frac{\partial w}{\partial I_{2}}\right)\\ \sigma_{T3}-\sigma_{T1}=2\left(\lambda_{3}^{2}-\lambda_{1}^{2}\right)\left(\frac{\partial w}{\partial I_{1}}+\lambda_{2}^{2}\frac{\partial w}{\partial I_{2}}\right) \end{array}$$

The relationship between actual stress and engineering stress is:

(7) $$\sigma_{i}=\frac{\sigma_{Ti}-\sigma_{Tj}}{\lambda_{i}}$$

where $\sigma_{i}$ is engineering stress, $\sigma_{Ti},\;\;\sigma_{Tj}$ is actual stress in different directions.

Through the relationship between the stress and strain of the rubber material, the parameters in the strain energy density function can be determined. This uses the uniaxial tensile test method or the biaxial tensile test method.

In the uniaxial tensile test, the force is applied in the main direction, $\sigma_{1}=\sigma$, and in the other two directions $\sigma_{2}=\sigma_{3}=0$. According to the principle of constant volume before and after deformation of silicone rubber material, $\lambda_{1}=\lambda$. According to formula (4), $\lambda_{2}^{2}=\lambda_{3}^{2}=\lambda^{-1}$. According to Equation (6), the stress difference in each direction can be obtained under uniaxial tension, rubber material stress and strain formula:

(8) $$\sigma_{1}\lambda_{1}=2\left(\lambda_{1}{}^{2}-\lambda_{2}{}^{2}\right)\left(\frac{\partial w}{\partial I_{1}}+\lambda^{-1}\frac{\partial w}{\partial I_{2}}\right)$$

$\sigma_{1}=\sigma ,\lambda_{1}=\lambda ,\;\lambda_{2}^{2}=\lambda_{3}^{2}=\lambda^{-1}$, substitution formula (8)

(9) $$\sigma =2\left(\lambda -\lambda^{-2}\right)\left(\frac{\partial w}{\partial I_{1}}+\lambda^{-1}\frac{\partial w}{\partial I_{2}}\right)=2\left[\left(1+\varepsilon \right)-{\left(1+\varepsilon \right)}^{-2}\right]\left[\frac{\partial w}{\partial I_{1}}+{\left(1+\varepsilon \right)}^{-1}\frac{\partial w}{\partial I_{2}}\right]$$

The mechanical properties of rubber elastomers depend on material parameters. In the uniaxial tensile experiment of rubber, the relationship between engineering stress σ and engineering strain ε can be measured using the uniaxial tensile experiment. According to the selection of different strain energy density function models, the corresponding material parameters of the strain energy density function can be determined using formula (9).

According to the theoretical analysis of the deformation basis of the rubber hyperelastic model, the second-order strain energy density function Yeoh model can be expressed as:

(10) $$w=C_{10}\left(\lambda^{2}+\frac{1}{\lambda^{2}}-2\right)+C_{20}{\left(\lambda^{2}+\frac{1}{\lambda^{2}}-2\right)}^{2}$$

The principal stress $\sigma_{i}$ along each coordinate axis is a function of $w,\varepsilon_{i}$ and the Lagrangian multiplier p. The specific function expression is formula (11).

(11) $$\sigma_{i}=\frac{\partial w}{\partial \lambda_{i}}-\frac{p}{\lambda_{i}}$$

When external air pressure is applied to the drive air cavity, the bottom limiting layer and the ring-shaped limiting structure will restrict the axial direction of the bottom of the driver; this can make the drive bend inward. $\lambda_{2}$ is drive joint radial elongation. The annular restraint structure can restrain the radial deformation of the drive joint; therefore, $\lambda_{2}=1$.

From formula (3), $\lambda_{1}\lambda_{2}\lambda_{3}=1$, $\lambda_{1}=\lambda ,\lambda_{2}=1,\lambda_{3}=\lambda^{-1}$.

From formula (11), we can combine the following equations:

(12) $$\{\begin{array}{l} \sigma_{1}=\frac{\partial w}{\partial \lambda_{1}}-\frac{p}{\lambda_{1}}\\ \sigma_{2}=\frac{\partial w}{\partial \lambda_{2}}-\frac{p}{\lambda_{2}}\\ \sigma_{3}=\frac{\partial w}{\partial \lambda_{3}}-\frac{p}{\lambda_{3}}=0 \end{array}$$

(13) $$p=2\lambda^{-2}\left[C_{10}+2C_{20}\left(\lambda_{1}^{2}+\lambda_{2}^{2}+\lambda_{3}^{2}-3\right)\right]$$

According to Equation (12), λ=1, $\sigma_{1}=\sigma_{2}$, λ>1, $\sigma_{1}>\sigma_{2}$, axial stress $\sigma_{1}$ is much larger than the circumferential stress $\sigma_{2}$, so take $\sigma_{1}$ as the only principal stress during the movement of the drive and express it as σ.

In order to study the relationship between the air pressure load of the bionic interphalangeal joint and the bending deformation angle, the cavity structure of the finger joint is the research object. When the finger is loaded by positive air pressure, the asymmetry of the cavity structure causes anisotropic bending and deformation of the finger joints. Throughout the bending process, $M_{p}$ is the resultant moment caused by the external positive pressure load on the inner surface of the air cavity. $M_{\theta }$ is the resultant impedance torque generated by the bottom, top and side walls of the driving joint. After the finger is bent and stabilized, the torque generated by the external load air pressure on the inner wall of the air cavity and the impedance torque generated inside the driver is in a balanced state:

(14) $$M_{p}=M_{\theta }$$

where $M_{P}$ is torque generated by external air pressure, $M_{\theta }$ is impedance torque generated inside the driver.

(15) $$M_{p}=2p\int_{0}^{\pi /2}\int_{0}^{r}\left(rsin\theta +d_{1}+d_{2}\right)rdrd\theta +2p\int_{0}^{d_{1}}r\left(y+d_{2}\right)d_{y}$$

where $M_{p}$ is the input air pressure to generate torque(N·mm), p is the air pressure (MPa), $d_{1}$ is the rectangular chamber height (mm), $d_{2}$ is the bottom thickness of the air cavity (mm), y is the vertical distance between the rectangular chamber and the upper surface of the bottom of the drive (mm), r is the inner diameter of air cavity (mm), θ is the single air cavity bending angle (rad).

When the finger is bent, the inner wall of the air cavity is deformed, and the internal resistance torque $M_{\theta }$ is generated after the finger joint is stabilized. There are three parts: the driver top impedance torque $M_{\sigma_{0}}$, the intermediate driver impedance torque $M_{\sigma_{1}}$ and the impedance torque of the bottom driver $M_{\sigma_{2}}$.

(16) $$M_{\theta }=M_{\sigma_{0}}+M_{\sigma_{1}}+M_{\sigma_{2}}$$

The axial stretch ratio of each part can be expressed as $\lambda_{\delta_{i}}$(i = 0, 1, 2)

(17) $$\{\begin{array}{l} \lambda_{\delta_{0}}=\frac{\left(r\;+\;\delta_{0}\right)sin\alpha \;+\;d_{1}\;+\;d_{2}\;+\;R}{R}=\frac{\theta \left(r\;+\;\delta_{0}\right)sin\alpha \;+\;d_{1}+d_{2}\;+\;L/4}{L/4}\\ \lambda_{\delta_{1}}=\frac{\delta_{1}\;+\;d_{2}\;+\;R}{R}=\frac{\theta \left(\delta_{1}\;+\;d_{2}\right)\;+\;L/4}{L/4}\\ \lambda_{\delta_{2}}=\frac{\delta_{2}\;+\;R}{R}=\frac{\theta \delta_{2}\;+\;L/4}{L/4} \end{array}$$

where L is joint air cavity length (mm), R is the radius of curvature (mm), $\lambda_{\delta_{i}}$ is elongation, $\delta_{i}$ is incremental displacement of section thickness (mm).

The resistance torque generated by the stretching of each layer of the drive around the center of rotation:

(18) $$\{\begin{array}{l} M_{\sigma_{0}}=\int_{0}^{\pi }\int_{0}^{d_{0}}\sigma_{\delta_{0}}\left(\left(r+d_{0}-\delta_{0}\right)sin\alpha +d_{1}+d_{2}\right)\left(r+d_{0}-\delta_{0}\right)d_{\delta_{0}}d_{\alpha }\\ M_{\sigma_{1}}=2\int_{0}^{d_{1}}\sigma_{\delta_{1}}\left(\left(r+d_{0}\right)\right)\left(d_{1}+d_{2}-\delta_{1}\right)d_{\delta_{1}}\\ M_{\sigma_{2}}=2\int_{0}^{d_{2}}\sigma_{\delta_{2}}\left(\left(r+d_{0}\right)\right)\left(d_{2}-\delta_{2}\right)d_{\delta_{2}} \end{array}$$

**Figure 7:**
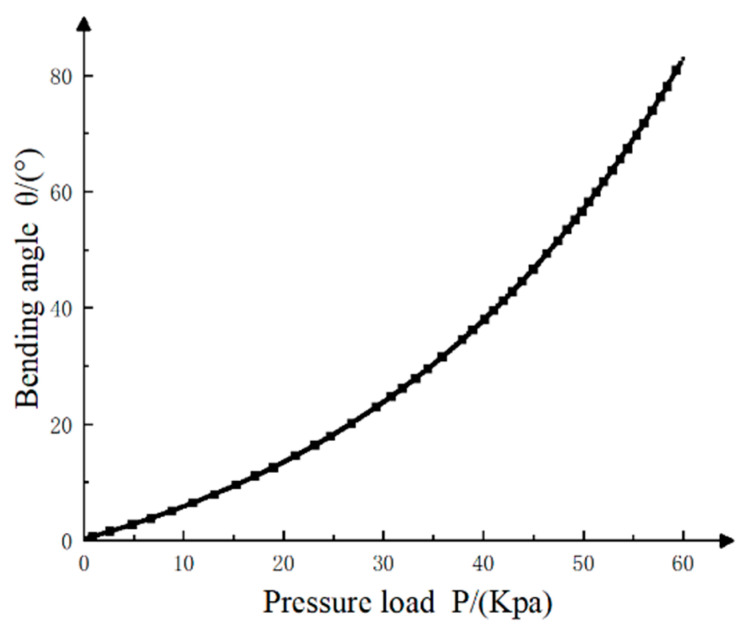
Relationship between air pressure and drive bending angle.

From formulas (14) and (16):$\;M_{p}=M_{\sigma_{0}}+M_{\sigma_{1}}+M_{\sigma_{2}}$. Through numerical integration, the relationship between the input air pressure P and the bionic finger drive angle θ can be obtained. Figure 7 show that the overall bending change of the driving joint shows a non-linear trend. When the external air pressure load is 60 Kpa, the bending angle of the proximal phalanx drive joint is approximately 84°.

### 3.2. Finite Element Simulation Analysis of Soft Driver

In order to verify the feasibility of the design, ABAQUS finite element analysis software was used to simulate and analyze the driving part of the soft finger.

The type of material constitutive model is the second-order Yeoh model. Boundary conditions are set to end fixed. The applied load is pressure (uniform inside the cavity, pressure gradually increases). The grid type is a quadratic grid.

**Figure 8:**
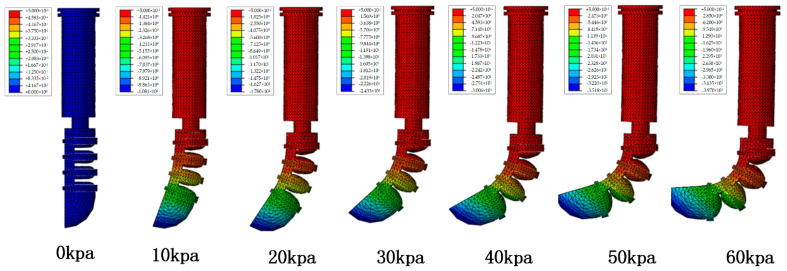
Simulation results of the bending of the distal phalanx of the thumb.

In the case of adding a flexible steel plate, Figure 8 shows that the software drives the joint bend to the limit layer. As the external air pressure increases, the bending angle of the finger drive joint tends to increase. Under an external load of 0.06 Mpa, the bending angle of the distal phalangeal joint is 95°. This analysis result can provide a theoretical basis for the follow-up bionic hand movement experiment.

**Figure 9:**
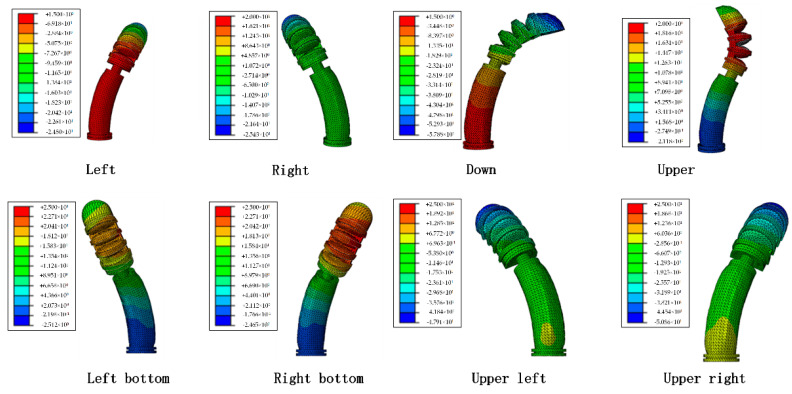
Thumb base joint simulation.

We performed a simulation analysis of the flexion of the soft thumb base joint and the distal phalanx joint. First, the external air pressure applied in the distal phalanx joint was fixed at 40 Kpa. Secondly, the external load on the base joint of the thumb was set, and the four air cavities were loaded separately. The simulation results are shown in Figure 9.

**Figure 10:**
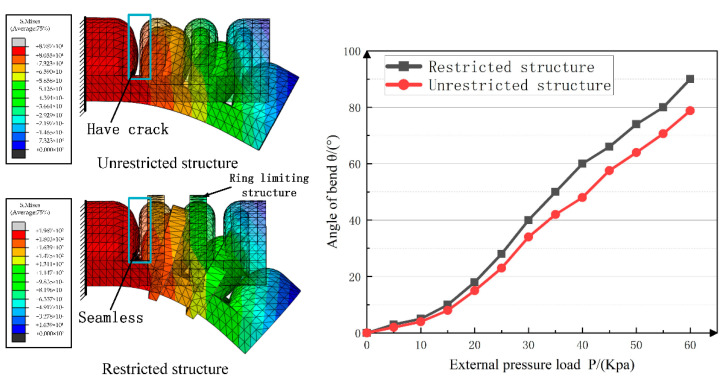
Comparison of simulation results of driving joints.

The middle phalanx joint structure was used as the research object to analyze and discuss the influence of the ring reinforcement on the performance of the driving joint. The simulation result is shown in Figure 10. The specific data description is shown in Table 2.

Under the same other conditions, the drive joints of the ring-shaped restriction structure were added, and the contact between the air chambers was more sufficient. Simulation analysis verified that the improved structure could improve the bending and deformation performance of the pneumatic joint.

## 4. Design of Bionic Gripping Hand Control System

**Figure 11:**
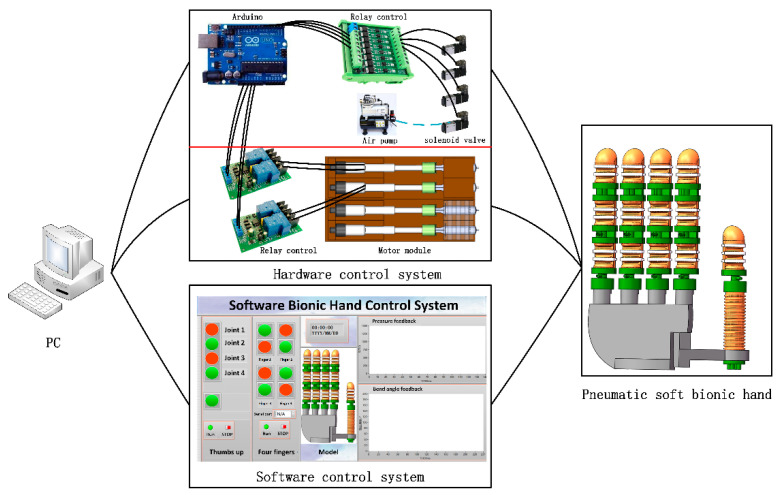
The overall scheme of the soft bionic hand control system.

The pneumatic soft bionic hand control system includes a hardware control system and soft control system. Figure 11 show that the hardware control system includes a pneumatic control loop system and sensor system. The soft control system includes the upper computer operation interface and the lower computer control program.

### 4.1. Design of Pneumatic Control System Based on Motor Micropump

The control system is mainly composed of the man-machine interactive control module and Arduino control center. Using the LabView control interactive interface input, the PC host computer sends the control command to the Arduino control center through the RS232 serial port protocol, and then the Arduino controller will receive the command analysis and send the control command to the motor control relay through the I/O port. In addition, the LabView control program collects the data fed back from the flexible bending sensor and the flexible pressure sensor to the Arduino in real-time, and the current status of the drive is visually displayed through the interface.

During the movement of the soft finger, the air pressure sensor module detects the data of the current soft-driven air cavity and compares it with the set target value. The forward and reverse rotation of the motor can be controlled to make the air pressure closer to the set value. The motor micro-pump pneumatic control system has a simple structure and can dynamically control the movement of the bionic hand.

### 4.2. Design of Bionic Hand Soft Control System Based on LabView

**Figure 12:**
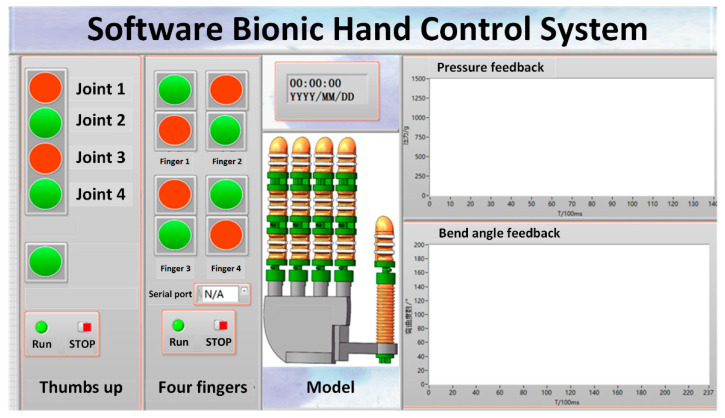
GUI control interface.

According to the moving target of the bionic hand, the design of the user interface includes manual control of the bionic finger-driven joints and a display of the experimental curve of the driving joint sensor. In manual mode, the user can independently control the air pressure load in each drive joint to complete the bending of the drive joint at different angles. In the overall control mode, the bionic finger makes corresponding coordinated motions according to the set motion mode. As shown in Figure 12, it adopts the GUI interface development module of LabView to design the online control interface of the bionic hand. The control system receives the command from the host computer through Arduino and transmits it to the driver.

## 5. Experimental Analysis

### 5.1. Soft Thumb Bending Performance Experiment

**Figure 13:**
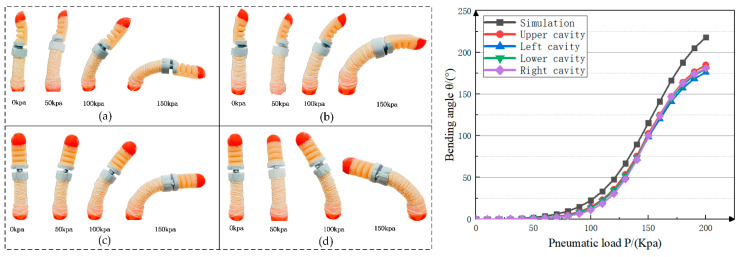
Thumb distal phalanx joint bending experiment: (**a**) External loads are applied to the upper chamber; (**b**) External loads are applied to the lower chamber; (**c**) External loads are applied to the left chamber; (**d**) External loads are applied to the right chamber.

First, the relationship between air pressure and the bending angle of each cavity of the soft thumb base joint was studied. Figure 13 shows different external air pressure cavities applied to the base of the thumb. After the drive was stabilized, the bending angle of the finger was recorded at 50 Kpa intervals.

From the experimental results, it was concluded that different external air pressure loads were applied to the driving joint cavity, and the obtained base joint bending angles were different, which is consistent with the soft simulation results.

**Figure 14:**
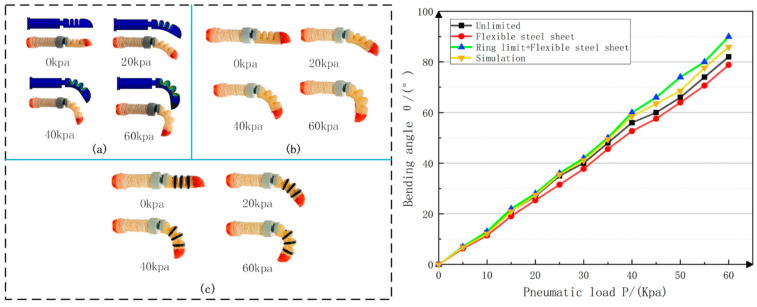
Thumb distal phalanx joint bending experiment: (**a**) no restrictions (Compare with simulation model); (**b**) only flexible steel plate; (**c**) confinement ring and flexible steel plate work together.

The bottom of the fixed thumb base joint was fixed on the test platform, and an external air pressure load of 0–60 Kpa was applied to the distal phalanx drive joint; the sampling interval was 20 Kpa. Figure 14 shows the comparison result.

The experimental results show that the addition of the flexible steel sheet at the bottom had an almost negligible influence on the bending of the soft-driven joint. In the air pressure range of 0–30 Kpa, the radial expansion was small. At this time, the addition of the ring-shaped reinforcement had little effect on the bending of the driving joint. In the range of 30–60 Kpa, the internal driving air cavity was under strong pressure, resulting in the problem of radial expansion. The addition of annular reinforcement restricted the radial expansion of the driving joint and converted it into a bending function of the driving joint. The maximum bending angle of the driving joint was increased by about 10°.

### 5.2. Soft Four-Finger Performance Analysis

**Figure 15:**
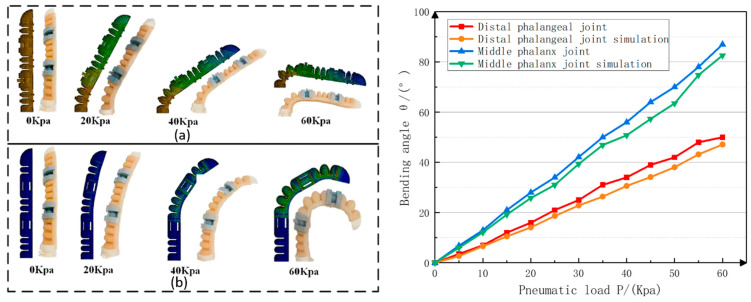
Middle finger bending experiment and simulation comparison of the bionic finger: (**a**) Bending near the phalanx; (**b**) The middle phalanx and the distal phalanx bend at the same time.

The analysis was in the range of 0–60 Kpa of external air pressure, and the sampling interval was 20 kpa. Figure 15 shows the comparison of experimental results and simulation analysis without constraining structures.

**Figure 16:**
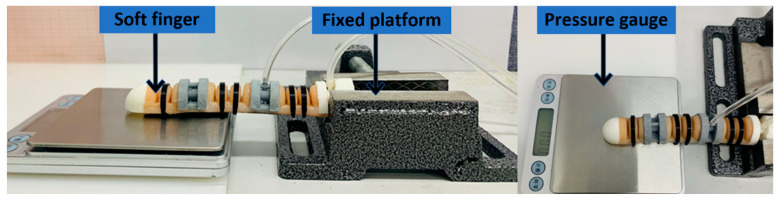
Soft fingertip output force experiment platform.

In order to clarify the output force characteristics of the soft finger, this section builds the test device shown in Figure 16 to measure the fingertip output force of different drive joints.

**Figure 17:**
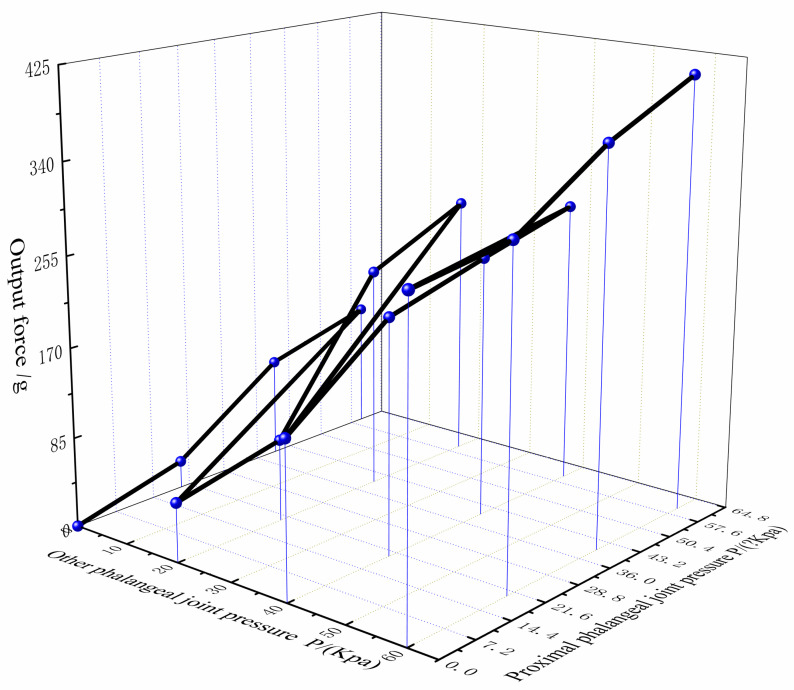
Drive joints to apply air pressure load output force together.

The experimental platform can make the soft finger touch the electronic dynamometer. The fingertips of the soft fingers were placed in the center of the electronic dynamometer. Figure 17 shows the load force performance of the fingertip when air pressure was applied to the finger actuation joint.

Statistical analysis of the acquired data shows that when only aerodynamic loads were applied to the proximal phalangeal joint, the maximum output force of the fingertip was about 3.06 N. When only the rest of the phalanx was applied with air pressure, the maximum output force of the fingertip was about 1.24 N. When the air pressure load was in the range of 40 Kpa–60 Kpa, the output force increase of the fingertip was obviously reduced. The main reason is that with the increase of air pressure, the soft finger will bend and warp, which makes the output force increase less. When the air pressure load was applied together in the finger drive joint, the maximum output load force of the fingertip was 4.11 N.

### 5.3. Bionic Hand Grasping Experiment and Humanoid Gestures

**Figure 18:**
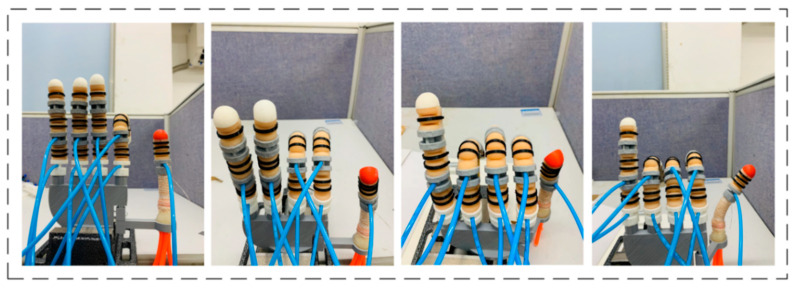
Bionic hand simulates human gesture operation.

Figure 18 show part of the gestures of the soft bionic hand simulating the human hand. Combined with the specific timing control scheme of the designed control system, different external air pressure loads were applied to the driving joints of each finger so that the fingers have different degrees of bending, and the gestures that can be completed are diversified. From the experimental results, it can be concluded that the soft-driven joints can be controlled independently, the middle phalange and the distal phalange joints can realize joint movement, and the synergy of different joints can realize the simulation of human gestures.

**Figure 19:**
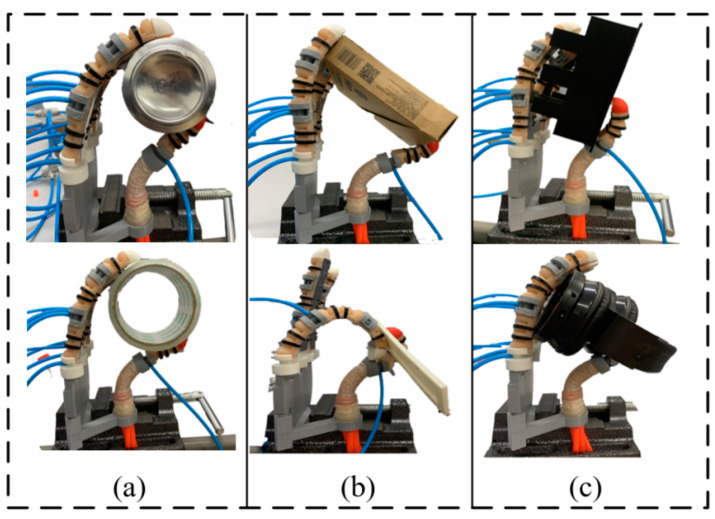
Soft bionic hand grasping experiment: (**a**) Cylinder; (**b**) Cuboid; (**c**) Irregular object.

Figure 19 show the experiment of grasping some common objects in daily life with the soft bionic hand. The grasping action was judged to be successful if the object did not fall during the grasping process.

## 6. Conclusions

In this paper, a new design of a pneumatic bionic hand with a rigid-flexible coupling structure was presented. In order to solve the problem of radial expansion and poor lateral bearing capacity of soft-drive joints, a design scheme with a composite annular restraint structure and an embedded flexible steel plate was proposed, which solved the problem of the difference between the radial deformation. Furthermore, in order to solve the problem of complex and integrated manufacturing and molding of the drive joint structure, we analyzed the structure and employed a layered manufacturing approach. We used 3D printing technology to design the molds and develop the bionic hands. We adopted the Yeoh second-order theoretical model to establish the mathematical deformation model of the bionic hand-driven air cavity, and the theoretical relationship between the external pressure load and the bending angle of the actuator was obtained. We used ABAQUS finite element simulation analysis software to simulate and analyze the motion of the finger drive joint. The design of the pneumatic control system of the motor micropump was carried out using Arduino to control the motor and other equipment. A visual interface based on Labviewwas developed to collect finger gestures in real-time. The experimental results show that the new design achieved high flexibility of the fingers and improved the lateral load-bearing performance of the fingers. It ascertained with the simulation of human gestures and the grasping task of conventional objects.

However, we only carried out the statics model, but the finger dynamics were not studied and analyzed. Next, we will solve the dynamic angle model of the finger actuated joint and analyze the bending behavior of the actuated joint during dynamic inflation and deflation. In further research and analysis, EMG signal sensors, visual sensors, etc., can be used to complete an intelligent grasping program, which can track and simulate human hand movements in real-time. This can be used to identify the grasped item and determine the required grasping force to complete the intelligent grasping program.

## Figures and Tables

**Table 1:** E60 Silicone performance parameter table.

Parameter	Numerical Range
Hardness (A)	20 ± 2
Tensile strength (MPa)	6.5
Tear strength (kN/m)	28 ± 2
Mixing ratio (A:B) ^1^	1:1
Room temperature curing time (h)	4

^1^ E60 silica gel is divided into two reagents, A and B. We need to mix A and B so that the silica gel can be cured.

**Table 2:** Drive joint simulation analysis test data.

Air Pressure	0 Kpa	10 Kpa	20 Kpa	30 Kpa	40 Kpa	50 Kpa	60 Kpa
No ring restriction	0°	4°	15°	34°	48°	63.5°	78.5°
limited	0°	5°	18°	40°	60°	74°	90°

## Data Availability

The study did not report any data.
